# Long non-coding RNA LINC00511 regulates the expression of microRNA-625-5p and activates signal transducers and activators of transcription 3 (STAT3) to accelerate the progression of gastric cancer

**DOI:** 10.1080/21655979.2021.1940611

**Published:** 2021-07-05

**Authors:** Ning Cui, Qinhui Sun, Hongjun Liu, Leping Li, Xiaobo Guo, Yulong Shi, Changqing Jing, Chengkun Qin, Yu Zhao

**Affiliations:** a Department of Gastroenterology, Renmin Hospital of Wuhan University, Wuhan, Hubei Province, China; b Department of Gastrointestinal Surgery, Shandong Provincial Hospital Affiliated to Shandong University, Jinan, Shandong Province, China

**Keywords:** Gastric cancer, LINC00511, miR-625-5p, STAT3

## Abstract

This study aimed to investigate the expression, biological function, and downstream mechanism of LINC00511 in gastric cancer (GC). In paired GC samples, LINC00511, miR-625-5p and STAT3 mRNA expression levels were detected by quantitative real-time polymerase chain reaction (qRT-PCR); STAT3 protein expression was detected by immunohistochemical (IHC). The gain-of-function and loss-of-function models were established, and the proliferative and migrative ability of GC cells were measured by CCK-8 and transwell assays, respectively. The regulatory relationship between miR-625-5p and LINC00511 or STAT3 was examined by bioinformatics analysis, luciferase reporter gene assay, qRT-PCR, and western blot. We reported that LINC00511 and STAT3 expressions in GC tissues and cell lines were observably up-regulated, while miR-625-5p expression was inhibited. High expression of LINC00511 could facilitate the proliferation and promote the migration of GC cells. miR-625-5p was proved to be a downstream target of LINC00511, and LINC00511 could induce the expression of STAT3 by inhibiting the expression of miR-625-5p. Additionally, knockdown of LINC00511 suppressed the growth and lung metastases of CRC cells in nude mice. In conclusion, LINC00511 promotes the GC cell proliferation and migration via targeting the miR-625-5p/STAT3 axis, implying that LINC00511 can function as a target for GC therapy.

## Graphical Abstract

**Figure:**
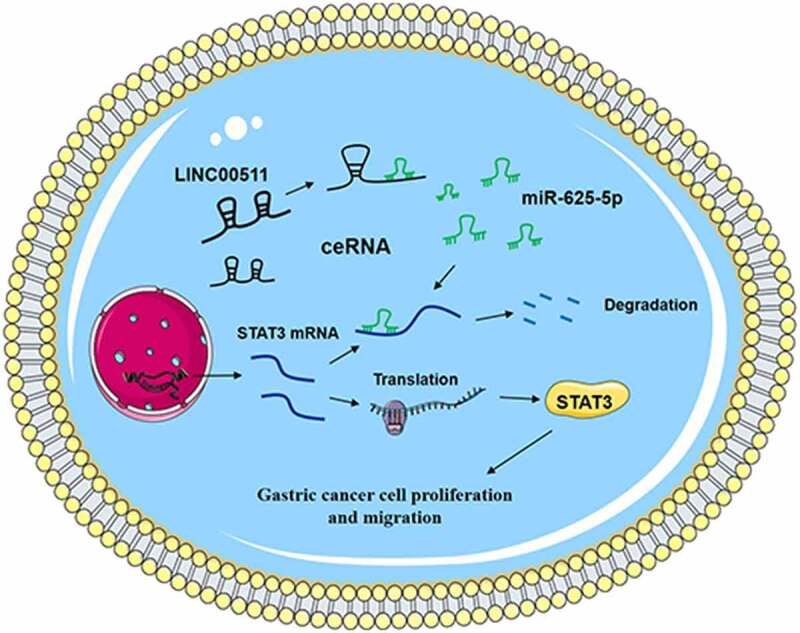

## 1. Introduction

Gastric cancer (GC) is one of the most common cancers in the world. The morbidity and mortality of GC in East Asia are higher, especially in Korea, Mongolia, Japan, and China [1,2]. The current strategies for treating GC mainly include surgical resection, adjuvant radiotherapy, and chemotherapy. However, GC remains a highly fatal disease [3]. In recent years, non-coding RNA (ncRNA) has become a new source of biomarkers and therapy targets in cancer research [4,5]. The in-depth study of the relationship between long non-coding RNA (lncRNA) and GC tumorigenesis will pave a bright way for GC diagnosis and treatment.

Long non-coding RNAs (lncRNAs), ncRNA molecules with about 200nt, are participants in diverse biological processes, such as cell proliferation, apoptosis, differentiation, inflammation, and angiogenesis [6]. LncRNA is abnormally expressed in many tumors. For example, lnc-BMP1-1 expression is suppressed in the tumor tissues of lung cancer patients, especially those with history of smoking, and the growth and migration of lung cancer A549 cells with lnc-BMP1-1 overexpression are inhibited [7]. Overexpression of lnc-MUC20-9 in bladder cancer cells leads to the reduced viability, colony formation, migration, invasion, and increased apoptosis [8]. LINC00511 is transcribed from chromosome 17q24.3 region, and it is up-regulated in tumors such as hepatocellular carcinoma, colorectal cancer, and GC [9–11].

Recognized as single-stranded small-molecule RNAs with about 20–22nt, miRNAs can modulate gene expression after transcription [12]. Increasing studies have shown that miRNAs take part in regulating cancer progression [13,14]. For example, miR-25-3p inhibition blocks the tumorigenesis of GC [15]. miR-1269b inhibits GC development via regulating methyltransferase-like 3 [16]. miR-625-5p is cleaved and generated by precursor RNA transcribed from human chromosome 14q23.3. Recent studies have reported that miR-625-5p is abnormally expressed in cancers, such as non-small cell lung cancer, cervical cancer, and glioma [17–19].

A recent study has reported that LINC00511 can promote the progression of GC by targeting miR-625-5p and up-regulating NFIX [20]. However, the mechanism by which LINC00511/miR-625-5p axis promotes the GC progression has not been clarified clearly. In this work, we aimed to investigate the role of LINC00511 in the GC progression and the underlying mechanism. We report that LINC00511, as a competitive endogenous RNA (ceRNA) of miR-625-5p, plays a cancer-promoting role in GC via targeting miR-625-5p, which in turn increases the expression of signal transducers and activators of transcription 3(STAT3).

## 2. Materials and methods

### 2.1 Tissue samples

Our research was endorsed by the Ethics Committee of Shandong Provincial Hospital Affiliated to Shandong University, and the Ethics Committee of Renmin Hospital of Wuhan University, and all procedures were performed according to Declaration of Helsinki. Cancer tissues and adjacent tissues of 50 patients with primary GC who underwent gastrectomy in Shandong Provincial Hospital Affiliated to Shandong University from December 2017 to June 2018 were collected. Before surgery, none of the patients received anti-tumor treatment, and all the patients signed the informed consents. All tissues were obtained during the surgery and frozen in liquid nitrogen at −196°C before use.

### 2.2 Cell lines, cell culture

The Chinese Academy of Sciences Cell Bank was the provider of human GC cell lines MKN28, BGC-823, MKN-45, MGC-803, SGC-7901 and normal gastric mucosal epithelial cell-line GES-1. These cells were cultured in Dulbecco Modified Eagle Medium (DMEM) (Invitrogen, Carlsbad, CA, USA) with 100 U/ml penicillin G, 100 μg/mL streptomycin (Gibco, Life Technologies, Carlsbad, CA, USA) and 10% fetal bovine serum (FBS, Gibco, New York, CA, USA). The cells were maintained in 5% CO_2_ at 37°C in an incubator. Besides, the medium was changed every 3 to 4 days.

### 2.3 Cell transfection

pcDNA empty vector (NC), pcDNA-LINC00511, siRNA normal control (si-NC), siRNAs against LINC00511 (si-LINC00511), miRNA control (miR-NC), miR-625-5p mimic and miR-625-5p inhibitor were purchased from GenePharma (Shanghai, China). MKN28 and MGC-803 cells were inoculated on a 24-well cell plate at 3 × 10^5^ cell/well, and cultured in 5% CO_2_ at 37°C for 24 h, and then transfected employing Lipofectamine® 3000 (Invitrogen, Carlsbad, CA, USA) according to the manufacturer’s instruction. The transfection efficiency was examined by quantitative real-time polymerase chain reaction (qRT-PCR).

### 2.4 qRT-PCR

Total RNAs in tissues or cells were extracted by TRIzol reagent (Invitrogen, Waltham, MA, USA). Nanodrop-spectrophotometer was used to detect the RNA concentration and purity. Based on the instructions, a PrimeScript RT Kit (Promega, Madison, WI, USA) was used to reversely transcribe 1 μg total RNA to synthesize its complementary DNA (cDNA). With cDNA as template, qRT-PCR was performed with a SYBR® Premix Ex Taq^TM^ kit (Takara, Otsu, Japan). GAPDH was the internal reference of LINC00511 and STAT3, and U6 was that of miR-625-5p. The primers were synthesized by RiboBio Co.,Ltd. (Guangzhou, China).

### 2.5 Immunohistochemistry

Streptomycin avidin-peroxidase (SP) method was used in immunohistochemistry. The tissue samples were prepared into paraffin sections. The sections were baked at 70°C and rehydrated with different gradients of ethanol, and then antigen retrieval was performed. Subsequently, H_2_O_2_ solution was added to cover the tissues in order to inactivate endogenous peroxidase at room temperature for 10 min, and then the sections were washed with PBS for 10 min. After that, anti-STAT3 antibody (1:200, ab119352, Abcam, Cambridge, UK) was added and the sections were incubated overnight at 4°C. Then the sections were rinsed with PBS three times, and the secondary antibody was added to incubate the section for 30 min at room temperature. After rinsing with PBS three times again, the sections were stained with DAB. Ultimately, the sections were observed and scored by two independent pathologists.

### 2.6 Cell counting kit-8 (CCK-8) assay

MKN28 and MGC-803 cells were inoculated into 96-well plates (3000 cells per well) and cultured for 1, 2, 3 and 4 days, respectively. On each day, 10 μL of enhanced CCK-8 solution (Dojindo, Kumamoto, Japan) was loaded into per well and incubated at 37°C for 1 h. Next, the absorbance of the cells was measured at 450 nm. Ultimately, the growth curve was plotted.

### 2.7 Transwell assay

Transwell migration assay was used to detect the cell migration. MKN28 and MGC-803 cells were trypsinized with 0.25% trypsin, centrifuged, resuspended and dispersed with serum-free medium, and the cell density of the cell suspension was adjusted to 5 × 10^5^ cells/mL. Subsequently, 200 μL of cell suspension was added into each upper chamber of the Transwell system (8 μm pore size, Corning, Beijing, China). At the same time, 400 μL of medium with 10% FBS was added into the lower chamber. After incubation at 37°C for 24 h, the cells that failed to migrate were removed from the upper chamber, and the cells which passed through the membrane were fixed with 4% paraformaldehyde for 10 min, stained with 0.5% crystal violet and washed by tap water. At last, the membrane was dried and cells were counted under an inverted microscope.

### 2.8 Dual-luciferase reporter gene assay

The targeted relationship between miR-625-5p and LINC00511 or the 3ʹ-untranslated region (3ʹ-UTR) of STAT3 was verified by a luciferase reporter gene assay. First, the wild type (WT) LINC00511 sequence or WT 3ʹ-UTR fragments from STAT3 mRNA containing predicted miR-625-5p binding sites were inserted into pmiRGLO dual-luciferase miRNA target reporter vector (Promega, Madison, WI, USA) to construct the report vector pmiRGLO-LINC00511-WT or pmiRGLO-STAT3-WT. Second, the putative binding site of miR-625-5p in the LINC00511 or STAT3 3ʹ-UTR was mutated by GeneArt™ Site-Directed Mutagenesis PLUS System (Thermo Fisher Scientific, Inc., Carlsbad, CA, USA). Third, the mutant (MUT) LINC00511 sequence or STAT3 3ʹ-UTR sequence was inserted into the pmiRGLO vector to construct the reporter vector pmiRGLO-LINC00511-MUT or pmiRGLO-STAT3-MUT. Fourth, the corresponding reporter vectors and miR-625-5p mimic or NC mimic were co-transfected into MKN28 cells and incubated for 48 h, and after that, the luciferase activity was subsequently evaluated by a Dual-Luciferase Reporter Assay System (Promega, Madison, WI, USA).

### 2.9 Western blot

Cells were harvested, lysed with pre-cooled RIPA lysis buffer (Beyotime Biotcchnology, Shanghai, China) and incubated on ice for 20 min. After that, the mixtures were centrifuged for 20 min at 4°C at the speed of 13,000 r/min. After centrifugation, the supernatant was collected, and the protein was then quantified by a BCA protein quantification kit (Beyotime, Shangha, China). Next, the protein concentration was adjusted. The protein was heated in boiling water for 5 min, and then subjected to SDS-PAGE and transferred to PVDF membrane. Next the PVDF membrane was blocked with 3% BSA for 1 h at room temperature. Subsequently, anti-STAT3 antibody (1: 1000, ab119352, Abcam, Cambridge, UK) and anti-GAPDH antibody (ab181602, 1:2000, Abcam, Cambridge, UK) were then used to incubate the membrane overnight at 4°C. Next, the membrane was rinsed 3 times with TBST. Subsequently, the diluted secondary antibody (Beyotime, Shangha, China, 1:2000) was loaded, and the membrane was incubated for 40 min at room temperature, and eventually the membrane was immersed in TBST 3 times. Ultimately, an electrochemical luminescence (ECL) kit (Tanon, Shanghai, China) was used to visualize the protein bands.

### 2.10 In-vivo assay

The 4-week-old female BALB/c nude mice were chosen for xenografts experiments. The mice were maintained in a specific pathogen-free facility. All procedures were approved by Animal Care and Use Committee of Renmin Hospital of Wuhan University. For the xenograft tumorigenesis assay, the mice were subcutaneously inoculated with MGC-803 cells stably transfected with sh-LINC00511 or sh-Vector in the right flank (1 x 10^7^ cells per mouse). The tumor volume was calculated according to the formula (length × width^2^/2) and measured every 7 days. After 28 days, the mice were sacrificed and tumors were collected, and the tumor weight was determined. For lung metastasis assay, MGC-803 cells stably transfected with sh-LINC00511 or sh-Vector were injected into the tail veins of the mice (1 x 10^7^ cells per mouse), and the mice were sacrificed 3 weeks after injection. The lungs were removed and fixed with 4% paraformaldehyde for 0.5 h. Then, tissue sections (4 µm) were prepared, and stained them with hematoxylin and eosin (H&E). Metastatic nodules of the lung were counted and evaluated under an optical microscope.

### 2.11 Statistical methods

Statistical analysis was subjected to SPSS 16.0 software (SPSS Inc., Chicago, IL, USA). All experiments were performed in triplicate, with the data expressed in the form of mean ± standard deviation (x ± s). The significance of the difference was evaluated by Student’s *t* test or one-way ANOVA. The survival analysis was performed employing the Kaplan–Meier method. The correlation analysis was analyzed by Pearson’s correlation analysis. Statistically, *P* < 0.05 is significant.

## 3. Results

### 3.1 The expression characteristics of LINC00511 and STAT3 in GC

**Figure 1.**
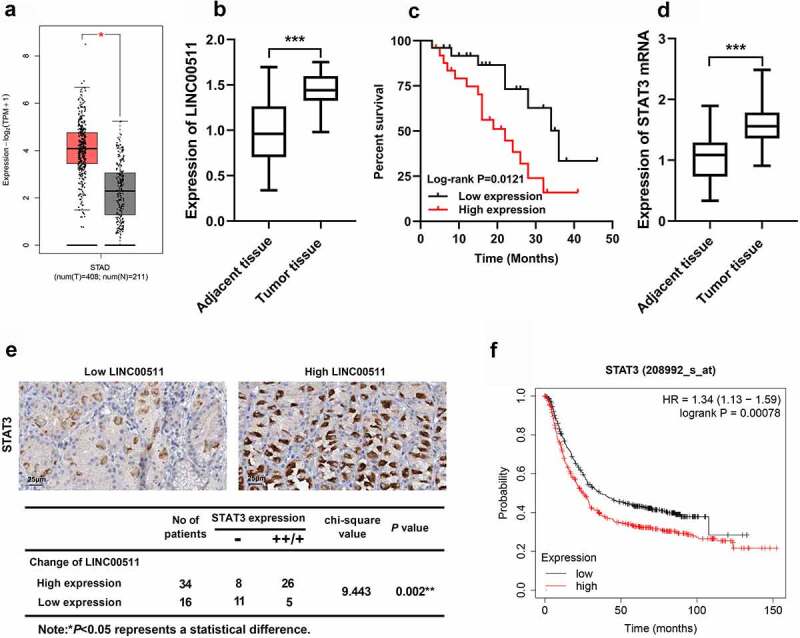
LINC00511 and STAT3 expression characteristics in GC. A. The expression of LINC00511 in GC tissues was analyzed in the GEPIA database. B. LINC00511 expression in GC tissues was detected by qRT-PCR. C. Kaplan−Meier analysis of the correlation between LINC00511 expression level and the overall survival time of GC patients. D. STAT3 expression in GC tissues was detected by qRT-PCR. E. Immunohistochemical method was used to analyze the expression of STAT3 in GC samples, and two representative figures were shown (left: low LINC00511, weakly positive stain of STAT3; right: high LINC00511, strongly positive stain of STAT3), and chi-square test was used to analyze the correlation between STAT3 expression and LINC00511 expression in cancerous tissues. F. The Kaplan-Merier Plotter database was used to analyze the relationship between STAT3 expression and the overall survival time of GC patients

To explore the role of LINC00511 and STAT3 in the GC progression, first of all, the expression characteristics of LINC00511 and STAT3 in GC were detected. Firstly, GEPIA database suggested that LINC00511 expressions in GC tissues were markedly increased compared with that in adjacent tissues (Figure 1a). Besides, we tested LINC00511 and STAT3 mRNA expressions in GC tissues by qRT-PCR. Consistent with our expectation, the results showed that the expression level of LINC00511 and STAT3 was raised in GC tissues (Figure 1b). The survival curve delineated that the high LINC00511 expression was associated with a worse prognosis of the patients with GC (Figure 1c). Additionally, the expression level of STAT3 mRNA in GC tissues was also up-regulated (Figure 1d). We further analyzed STAT3 protein expression by immunohistochemistry. It showed that the positive staining of STAT3 in GC tissues was positively correlated high expression of LINC00511 (Figure 1e). In addition, the Kaplan–Merier plotter database showed that patients with STAT3 high expression had shorter survival time (figure 1f).

*P < 0.05, **P < 0.001, and ***P < 0.001.

### 3.2 LINC00511 regulates the proliferation and migration of GC cells in vitro

**Figure 2.**
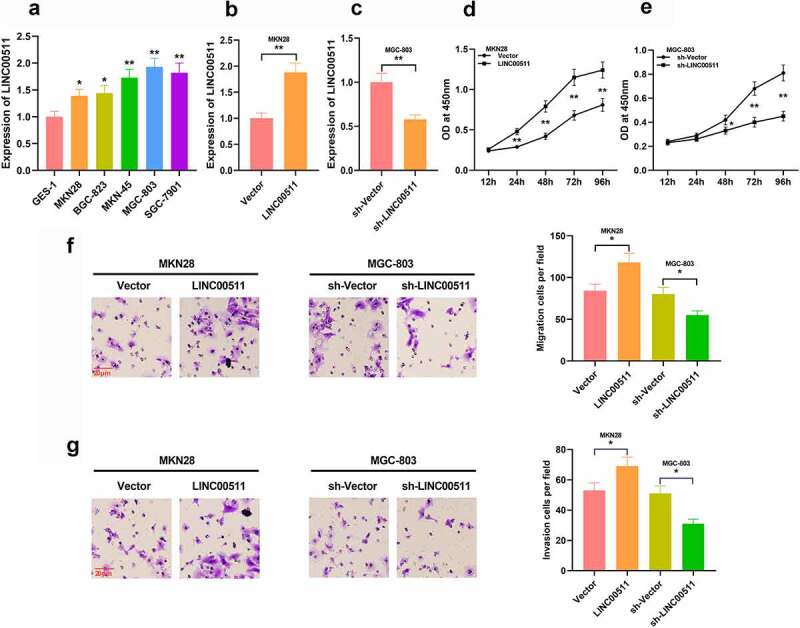
Effects of LINC00511 on GC cell proliferation, migration and invasion. A. qRT-PCR was adopted to detect LINC00511 expressions in GC cells and GES-1 cell line. B-C. qRT-PCR was used to detect the expression of LINC00511 after overexpression and knockdown of LINC00511. D-E. CCK-8 assay was used to detect the cell proliferation after overexpression and knockdown of LINC00511. F-G. Transwell assay was used to detect the migrative and invasive abilities of GC cells after overexpression and knockdown of LINC00511

To figure out the function of LINC00511 in GC, we examined LINC00511 expressions in GC cells, and qRT-PCR depicted that LINC00511 was elevated in GC cells in comparison to that of normal gastric mucosal epithelial cells (Figure 2a). We constructed LINC00511 overexpression and knockdown cell models in MKN28 cells with the lowest expression of LINC00511 and in MGC-803 with the highest expression of LINC00511, respectively; meanwhile, the transfection efficiency was validated by qRT-PCR (Figure 2b and c). Through CCK-8 assay, it was demonstrated that high expression of LINC00511 significantly promoted the proliferation of MKN28 cells, and inhibition of LINC00511 promoted the proliferation of MGC-803 cells (Figure 2d and e). Consistently, transwell assay indicated that overexpression of LINC00511 facilitated the migration and invasion of MKN28 cells, whereas inhibition of LINC00511 worked oppositely in MGC-803 cells (Figure 2f and g). In addition, the same results were observed in MGC-803 cells with LINC00511 overexpression and MKN28 cells with LINC00511 knockdown. These results indicate that LINC00511 can work as a cancer-promoting lncRNA in GC.

* *P* < 0.05, ** *P* < 0.01, and *** *P* < 0.01.

### 3.3 LINC00511 directly targets miR-625-5p

**Figure 3.**
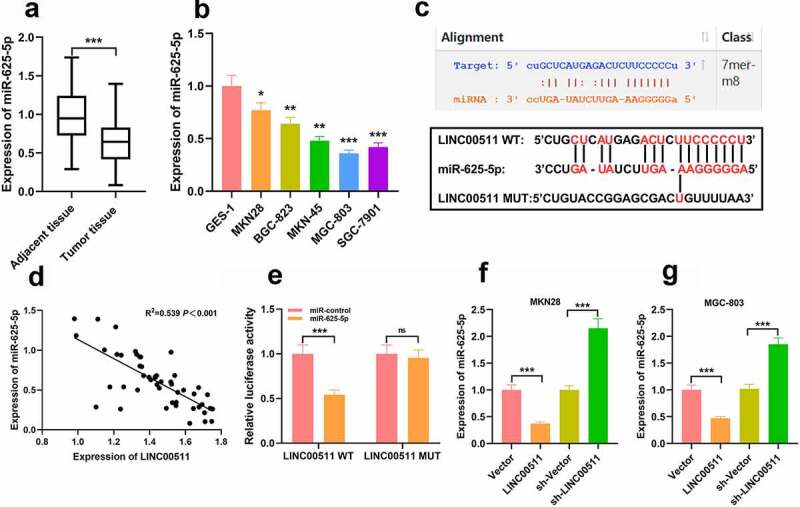
There is a targeted relationship between LINC00511 and miR-625-5p. A-B. qRT-PCR was used to detect the level of miR-625-5p in GC tissues and cell lines. C. Through bioinformatics analysis, it was found that LINC00511 contained a potential binding site for miR-625-5p. D. Pearson’s correlation analysis was used to detect the correlation between LINC00511 and miR-625-5p in tissues. E. The interaction between LINC00511 and miR-625-5p was validated by dual-luciferase reporter gene assay. F-G. qRT-PCR was used to detect the expression of miR-625-5p after overexpression and knockdown of LINC00511 in MKN28 and MGC-803 cells

To future investigate the mechanism of LINC00511 involved in GC progression, we examined miR-625-5p levels in GC tissue and cells, and the results showed that miR-625-5p was remarkably declined in GC tissues and cells (Figure 3a and b). Next, we adopted the bioinformatics analysis tool StarBase V2.0 to screen out potential downstream targets of LINC00511. As shown (Figure 3c), LINC00511 contained a potential conserved binding site for miR-625-5p. In addition, Pearson’s correlation analysis denoted that LINC00511 expression in GC tissues was negatively correlated with miR-625-5p expression (Figure 3d). To further validate the binding relationship between the above two molecules, we conducted a dual luciferase reporter gene assay, and it uncovered that miR-625-5p mimics could repress the luciferase activity of the luciferase reporter containing LINC00511-wt, but exerted no significant effect on that of the LINC00511-mut vector (Figure 3e). This implied that LINC00511 can interact with miR-625-5p. In addition, qRT-PCR results showed that overexpression of LINC00511 can inhibit miR-625-5p expression in MKN and MGC803 cells, while knocking down LINC00511 has the opposite effect in MKN and MGC803 cells (Figure 3f and g).

ns *P* > 0.05, * *P* < 0.05, ** *P* < 0.01, and *** *P* < 0.01.

### 3.4 Effects of miR-625-5p on the proliferation and migration of GC cells

**Figure 4.**
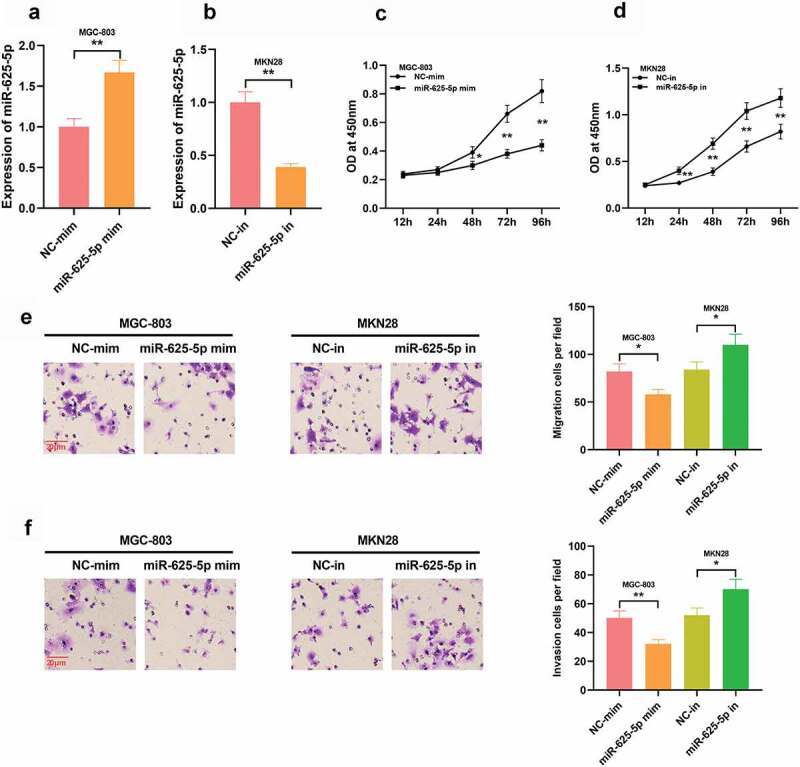
Effects of miR-625-5p on GC cell proliferation, migration and invasion. A-B. qRT-PCR was used to detect the expression of miR-625-5p after transfection of miR-625-5p mimics and inhibitors. C-D. The proliferation of cells transfected with miR-625-5p mimic and inhibitor was detected by CCK-8 method. E-F. Transwell assay was used to detect the migration and invasion of GC cells transfected with miR-625-5p mimic and inhibitor

To investigate the effect of miR-625-5p on GC cell proliferation and migration, we constructed miR-625-5p high and low expression cell models in GC cell lines MGC-803 and MKN28, respectively, with qRT-PCR validating the transfection efficiency (Figure 4a and b). CCK-8 assay showed that high expression of miR-625-5p significantly impeded the proliferation of MGC-803 cells, and inhibition of miR-625-5p promoted the proliferation of MKN28 cells (Figure 4c and d). It was also proved that miR-625-5p overexpression impeded the migration and invasion of MGC-803 cell, while inhibition of miR-625-5p worked oppositely in MKN28 cells (Figure 4e and f). These data suggest that miR-625-5p may be a tumor-suppressor in GC.

* *P* < 0.05, and ** *P* < 0.01.

### 3.5 STAT3 is the downstream target of miR-625-5p

**Figure 5.**
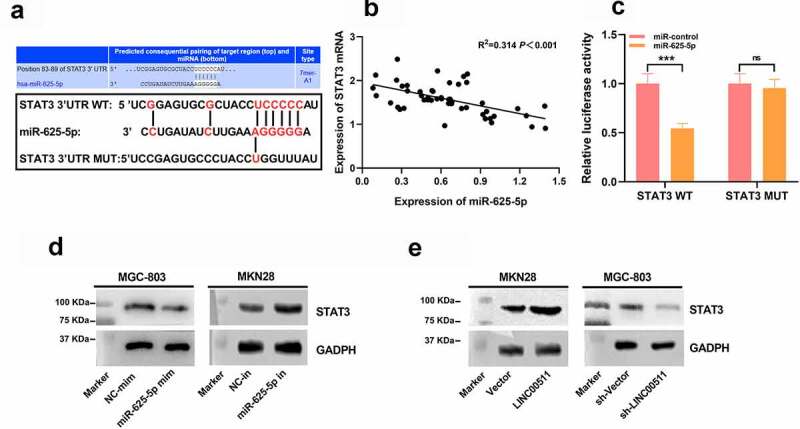
STAT3 is the functional target of miR-625-5p. A. Through StarBase database, it was found that the 3ʹUTR of STAT3 contained a potential binding site of miR-625-5p. B. Pearson’s correlation analysis was used to detect the correlation between STAT3 expression and miR-625-5p expression in GC tissues. C. Dual-luciferase reporter gene assay was used to validate the relationship between STAT3 and miR-625-5p. D-E. Western blot was used to detect the expression of STAT3 in GC cells transfected with miR-625-5p mimic and inhibitor, and the expression of STAT3 in GC cells transfected with LINC00511 overexpression and knockdown

To work out the mechanism of action of miR-625-5p in GC, we searched the StarBase database, screened out miR-625-5p candidate targets, and found that the 3ʹUTR of STAT3 contained a binding sequence for miR-625-5p (Figure 5a). It indicated that miR-625-5p may affect the proliferation and invasion of GC cells by repressing STAT3. Pearson’s correlation analysis denoted that miR-625-5p and STAT3 mRNA expressions were in negative correlation in GC tissues (Figure 5b). To elucidate the binding relationship between miR-625-5p and STAT3, a dual luciferase reporter assay was performed, it depicted that the miR-625-5p mimics could repress the luciferase activity of the luciferase reporter containing STAT3-wt, but no demonstrable change was observed on that of the STAT3-mut vector (Figure 5c). Western Blot assay depicted that transfection of miR-625-5p mimics down-regulated STAT3 expression, and transfection of miR-625-5p inhibitors promoted STAT3 expression; while overexpression of LINC00511 significantly elevated STAT3 expression, and knocking down LINC00511 worked oppositely (Figure 5d and e). These results indicate that in GC, STAT3, as a target of miR-625-5p, is positively modulated by LINC00511.

ns *P* > 0.05, and *** *P* < 0.001

### 3.6 LINC00511 affects the progression of GC via targeting miR-625-5p

**Figure 6.**
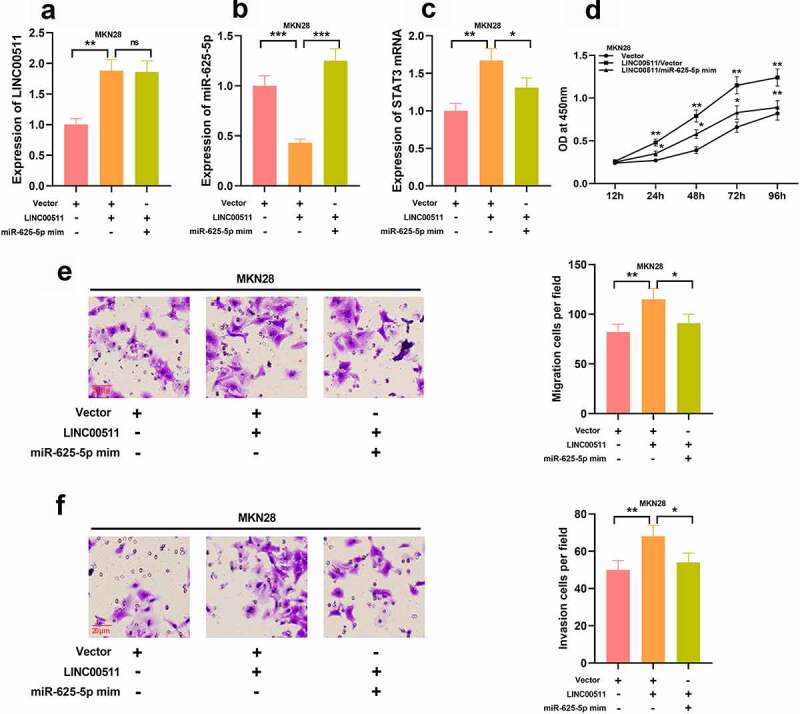
LINC00511 modulates the proliferation, migration and invasion of GC cell through regulating miR-625-5p. A-C qRT-PCR was used to detect the expression of LINC00511, miR-625-5p and STAT3 mRNA after transfection of miR-625-5p mimic into MKN28 cells with LINC00511 overexpression. D. CCK-8 method was used to detect the cell proliferation after transfection of miR-625-5p mimic into MKN28 cells with LINC00511 overexpression. E. MiR-625-5p mimic was transfected into MKN28 cells with LINC00511 overexpression, and the migration and invasion of the cells were detected by transwell assay

To explore how LINC00511/miR-625-5p works in regulating the proliferation and migration of GC cells, we transfected miR-625-5p mimics into MKN28 cells with LINC00511 overexpression (Figure 6a–C). We further examined the proliferation and migration of MKN28 cells, and CCK-8 and transwell experiments showed that the transfection of miR-625-5p mimics greatly reversed the proliferation, migration, and invasion of MKN28 cells induced by LINC00511 overexpression (Figure 6d–F). These results imply that the function of LINC00511 is partly dependent on its regulatory function on miR-625-5p.

* *P* < 0.05, and ** *P* < 0.01.

### 3.7 LINC00511 knockdown suppresses the tumor growth and lung metastasis of GC in vivo

**Figure 7.**
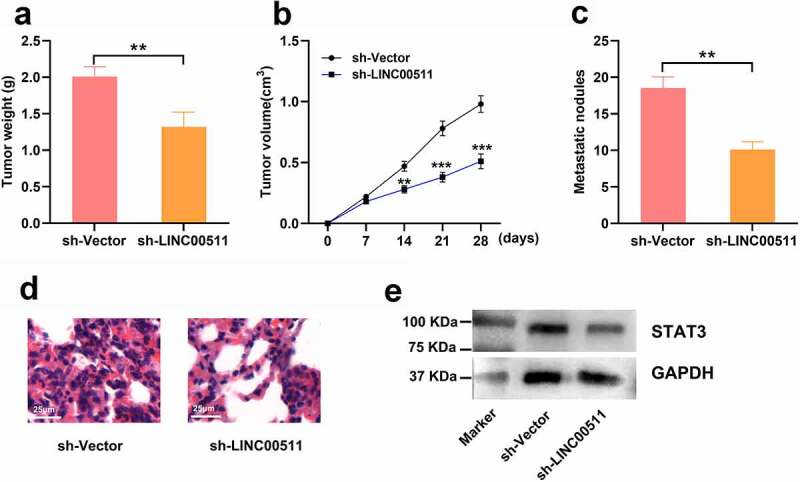
LINC00511 knockdown suppresses the tumor growth and lung metastasis of GC cells in vivo. A. MGC-803 cells with LINC00511 knockdown or the control cells were inoculated into the mice subcutaneously, respectively, and after the mice were sacrificed, the the tumor weight of the mice was measured (n = 5 in each group). B. Tumor volume of the mice in both groups was measured every 7 days (n = 5 in each group). C. MGC-803 cells with LINC00511 knockdown or the control cells were injected into the mice via tail vain, respectively, and after the mice were sacrificed, the number of metastatic nodules in the lung tissues of the mice was examined (n = 5 in each group). D. H&E staining was used to evaluate the metastasis of MCG-803 cells in the lung tissues sections (scale bar = 25 μm). E. Western blot assay was used to detect the expression of STAT3 in the tumor tissues of both groups

To investigate the effect of LINC00511 on GC growth and lung metastasis in vivo, we conducted xenograft nude mice models. The results showed that mice injected with MGC-803 cells with LINC00511 knockdown showed a reduction in tumor weight and volume compared with that of sh-Vector group (Figure 7a and b). Moreover, LINC00511 knockdown markedly reduced the formation of metastatic nodules in the lung tissues of the mice (Figure 7c and d). Also, the STAT3 protein expression showed a reduction in the tumor tissues of the mice in sh-LINC00511 group (Figure 7e). These data further support that knockdown of LINC00511 inhibits the tumor growth and metastasis of GC cells.

** *P* < 0.01, and *** *P* < 0.01.

## 4. Discussion

GC is one of the major disease burdens in China. It is estimated that in 2015, there are about 697,000 new cases of GC and about 500,000 deaths in China [21]. In recent years, the significance of lncRNA in GC has been increasingly recognized. For example, in *Helicobacter Pylori* infection, lncGNAT11 impedes the proliferation and invasion of GC cells through regulating the Wnt/β-catenin pathway [22]. The high expression of lncRNA HOTAIR is related to the poor prognosis of GC patients [23]. In this study, we demonstrated that LINC00511 expression was markedly elevated in GC tissues and cell lines. Additionally, functional experiments indicated that LINC00511 could promote the malignant biological behaviors of GC cells in vitro and in vivo. These data indicate that LINC00511 exerts a cancer-promoting effect in GC, which is consistent with the previous report [20].

Accumulating evidence supports that miR-625-5p serves as a tumor suppressor. For example, miR-625-5p modulates the glycolysis status of melanoma cells to repress the cancer progression by regulating PKM2 [24]. It also restrains the proliferation and increases apoptosis of non-small cell lung cancer cells [18]. Our work proved that miR-625-5p was lowly expressed in GC tissues and cell lines, and overexpression of miR-625-5p could inhibit the proliferation and invasion of cancer cells. These data validate that miR-625-5p is a tumor suppressor in GC.

STAT3 belongs to the family of signal transduction and transcription activators, which is involved in regulating multiple biological behaviors. After being activated, STAT3 is translocated to the nucleus, and it bands with DNA, and participates in signal transduction. STAT3 participates in the pathogenesis of various malignancies, including melanoma, prostate cancer and colonic cancer [25–27]. The excessive activation of STAT3 (p-STAT3) has been confirmed in different human tumors [28]. It is reported that the transcription and translation levels of STAT3 in GC tissue are markedly higher than those in normal tissues adjacent to cancer, and p-STAT3 level was associated with the differentiation status of GC [29]. In addition, STAT3 can target oncogenic factors and promote the proliferation and metastasis of GC cells [30]. In this work, it was demonstrated that in comparison with that of normal gastric tissue, STAT3 expression in GC tissue was greatly increased. Besides, the expression of STAT3 was positively correlated with LINC00511, and STAT3 was a downstream molecule of miR-625-5p. Our demonstrations imply that miR-625-5p can suppress the progression of GC by repressing STAT3 signal pathway.

ceRNA mechanism has drawn a lot of attention in cancer biology in recent years. In this mechanism, lncRNAs are described as competitors to mRNAs in binding with miRNAs, which facilitate the translation of mRNA. For example, LINC00612 is a ceRNA for miR-214-5p, and it can indirectly up-regulate SOX4 to promote the proliferation, migration, invasion and epithelial-mesenchymal transition of osteosarcoma cells [31]. LINC00461/miR-30a-5p axis regulates the progression of non-small cell lung cancer by modulating ZEB2 [32]. In GC, it is reported that LINC00265 promotes GC progression by regulating the miR-144-3p/CBX4 axis [33]. Additionally, lncRNA HCP5 can regulate proliferation, invasion, migration, and promotes apoptosis via miR-299-3p/SMAD5 axis in GC cells [34]. Inspired by the ceRNA regulatory network, we made a hypothesis that LINC00511 could also be a ceRNA. In the present work, we explored the interactions among LINC0051, miR-625-5p and STAT3. Bioinformatics analysis and dual-luciferase reporter gene assay predicted and verified that miR-625-5p could specifically bind to LINC00511 and the 3ʹUTR of STAT3, and our data also showed that miR-625-5p had a negative correlation with the expressions of LINC00511 and STAT3, and there was a positive correlation between the expressions of LINC00511 and STAT3. Importantly, miR-625-5p overexpression could counteract the effects of LINC00511 on promoting the malignant phenotypes of GC cells. Collectively, these data validate that LINC00511 is a ceRNA for miR-625-5p, and it promotes GC progression partly by regulating miR-625-5p/STAT3 axis.

## 5. Conclusion

In summary, LINC00511 promotes the GC cell proliferation and metastasis in vitro and in vivo via regulating miR-625-5p and STAT3, suggesting that LINC00511 exhibits oncogenic properties in GC progression. These findings bring new insights into the mechanism of GC progression, and provide novel ideas for GC diagnosis and therapy. However, larger number of clinical samples from multiple medical centers are needed to further evaluate the potential of LINC00511 as a prognostic biomarker.

## Supplementary Material

Supplemental Material

## Data Availability

The data used to support the findings of this study are available from the corresponding author upon request.

## Highlight

1. LINC00511 is up-regulated in gastric cancer tissues, and it is associated with poor prognosis of the patients.
2. LINC00511 promotes the migration and invasion of gastric cancer cells.
3. LINC00511 can induce the expression of STAT3 by inhibiting the expression of miR-625-5p.
