# Supplementary material for: Long non-coding RNA LINC00511 regulates the expression of microRNA-625-5p and activates signal transducers and activators of transcription 3 (STAT3) to accelerate the progression of gastric cancer
Source: Bioengineered. 2021 Jul 5;12(1):2915–27. doi: 10.1080/21655979.2021.1940611 (PMC8806821; doi:10.1080/21655979.2021.1940611)
Supplement: Supplemental Material [file KBIE_A_1940611_SM4382.zip › supplementary/Supplementary Figure.doc]

**Supplementary Figure 1 The LINC00511 expression profile across all tumor samples in TCGA.**

**Supplementary Figure 2 Effects of LINC00511 on GC cell proliferation, migration, and invasion.**

A-B qRT-PCR was used to detect the expression of LINC00511 after overexpression and knockdown of LINC00511. C-D CCK-8 assay was used to detect the cell proliferation after overexpression and knockdown of LINC00511. E-F: Transwell assay was used to detect the migration and invasion abilities of GC cells after overexpression and knockdown of LINC00511.

* *P* <0.05, ** *P* <0.01, *** *P* <0.01.
