# Supplementary material for: Long non-coding RNA LINC00511 regulates the expression of microRNA-625-5p and activates signal transducers and activators of transcription 3 (STAT3) to accelerate the progression of gastric cancer
Source: Bioengineered. 2021 Jul 5;12(1):2915–27. doi: 10.1080/21655979.2021.1940611 (PMC8806821; doi:10.1080/21655979.2021.1940611)
Supplement: Supplemental Material [file KBIE_A_1940611_SM4382.zip › supplementary/Supplementary materials.doc]

**Supplementary materials**

Figure 5D MGC-803 STAT3

Figure 5D MGC-803 GAPDH

Figure 5D MKN28 STAT3

Figure 5D MKN28 GAPDH

Figure 5E MKN28 STAT3

Figure 5E MKN28 GAPDH

Figure 5E MGC-803 STAT3

Figure 5E MGC-803 GAPDH

Figure 7E STAT3

Figure 7E GAPDH
